# The role of sexting in couple wellbeing for Italian women during the second wave of the COVID-19 pandemic

**DOI:** 10.3389/fpsyg.2023.1105556

**Published:** 2023-03-08

**Authors:** Rubinia Celeste Bonfanti, Maria Garro, Gioacchino Lavanco, Stefano Ruggieri

**Affiliations:** ^1^Department of Psychology, Educational Science and Human Movement, University of Palermo, Palermo, Italy; ^2^Faculty of Human and Social Sciences, Kore University of Enna, Enna, Italy

**Keywords:** COVID-19, women, sexting, sexual satisfaction, couple, wellbeing

## Abstract

The social isolation due to the COVID-19 pandemic had an impact on the sexuality and quality of life of people around the world. A particularly negative effect was detected on women’s sexual health. As a consequence, many women began to use social media not only to stay in touch with their social networks, but as a way of maintaining sexual contact. The main aim of this research is to observe the positive effects of sexting in women’s wellbeing as a strategy to manage the negative effects of a condition of forced isolation. We collected all our data between November 2020 and March 2021 during a period of strict restrictions in Italy due to the second wave of the COVID-19 pandemic. In Study 1, the relationship between loneliness, sexting behaviors, and sexual satisfaction was tested on 312 adult women. The results showed the mediator role of motivation for sexting in the relationship between loneliness and sexual satisfaction. In Study 2, 342 adult women were organized into two groups (women who had sexting at least once during the second wave of the pandemic = 203, and women who did not have sexting during the pandemic = 139) and were assessed on a couple’s wellbeing (intimacy, passion, commitment, and couple satisfaction) and electronic surveillance. The results show that women who had sexting during isolation had higher scores on intimacy, passion, couple satisfaction, and electronic surveillance. These findings suggest the important role of sexting as an adaptive coping strategy during particular conditions of social isolation.

## Introduction

Over the last few years, due to the coronavirus disease (COVID-19), the subjective wellbeing of many citizens around the world has been severely threatened. Actually, this condition is not strictly connected with the health problems associated with the pandemic or the fear of contracting the virus, but rather with the restrictions on individual freedom that all governments of the world adopted to minimize the spread of the virus. Many of these strategies were based on social distancing and limiting the mobility of the population.

A growing number of studies have been analyzing the negative psychological consequences of social isolation on the individual’s wellbeing and the impact of separation on social life, with particular reference to community, friends and couples’ relationships (Sikali, 2020; Gan and Best, 2021; Pietromonaco and Overall, 2022). Researchers have also found that social isolation increases level of stress (Fitzpatrick et al., 2020; Sterina et al., 2022), social anxiety (Wang et al., 2020), depression (Fischer et al., 2020), loneliness (Ruggieri et al., 2021a), and causes low levels of life satisfaction and psychological wellbeing (Satici et al., 2020; Zhang et al., 2020).

The experience of isolation due COVID-19 drove people to remain more socially connected *via* information and communication technology than before (Statista, 2022). This is not surprising because social media offered a unique opportunity to keep in touch with one’s own social network (Boursier et al., 2020). In this situation, many people have been driven online also in order to preserve their emotional relationships (Lindberg et al., 2020; Pietromonaco and Overall, 2022). Moving intimate relationships online has been a trend that has been going on for some time, but which has taken on much more significant tones and dimensions with the pandemic (Ruggieri et al., 2013; Burleson et al., 2022; Maes and Vandenbosch, 2022).

Existing research has found that traumatic events might lead to the deterioration of relationship quality and intimacy (Cohan and Cole, 2002; Marshall and Kuijer, 2017). For example, during the pandemic, a fear of COVID-19 infection generated distress with regard to couple intimacy, which altered sexual dynamics, especially for partners who did not live in the same home. Consequently, the intimacy and sexual life relationship of non-cohabiting couples was affected by isolation and went through a sharp change due to the pandemic. Isolation had a negative impact in terms of couple and sexual relationships, due to the difficulty of finding moments for intimacy (Lehmiller et al., 2020; de Oliveira and Carvalho, 2021). This situation has an impact on the increase in autoerotic sexuality, cybersex, cyber-pornography use, and sexting (Ibarra et al., 2020; Li et al., 2020). All these alternative sexual activities helped in managing the stress arising in a couple’s daily life, during the pandemic, representing a possible strategy of relief or compensation for the sense of loneliness (Uzieblo and Prescott, 2020).

Research has shown that the pandemic has had a particularly negative effect on women’s sexual health (Carvalho and Pascoal, 2020). A systematic review conducted during COVID-19 outbreak showed a deterioration of their sexual function, with a decline in sexual satisfaction (de Oliveira and Carvalho, 2021). Eleuteri et al. (2022) found that anxiety, stress and depressive symptoms due to the pandemic influenced a decrease in sexual desire for women. Previous studies also indicated that psychological distress had a negative impact, mostly on women’s sexuality (Dèttore et al., 2013; Kalmbach et al., 2014). An increase in household chores, the impossibility of having family support (for example nannies or housekeepers), and the little time devoted to the sexual sphere might also explain the decrease in sexual satisfaction for women during the COVID-19 pandemic (Craig and Churchill, 2020; Collins et al., 2021; De Rose et al., 2021).

In the wake of these literature suggestions, the main aim of the present research is to understand the role of sexting in women’s wellbeing and sexual satisfaction during the second wave of the COVID-19 pandemic.

### Sexting and sexual satisfaction during COVID-19 pandemic

Several researchers have studied the relationship between the inhibition of sexual desire and specific negative mood states emerging from the pandemic, such as anxiety, sense of loneliness, and the decrease in personal sexual satisfaction (Pascoal et al., 2021). This is probably one of the reasons why a decrease in sexual activity was found in research into couples in the United States (Hensel et al., 2020) and the United Kingdom (Jacob et al., 2020). If on the one hand psychological implications due to COVID-19 rendered the implementation of the sexual act less desirable, on the other hand government rules also reduced the possibility of maintaining sexual relationships because of enforced isolation.

Many studies have shown the effects of the pandemic and social isolation in women’s sexual satisfaction (de Oliveira and Carvalho, 2021). One of the difficulties faced by woman during confinement was that sexual wellbeing was considered non-essential, and sexual medication services were reduced, affecting women disproportionately (Hussein, 2020; Shindel and Rowen, 2020). For example, Endler et al. (2020) highlighted the fact that sexual and reproductive health care in various countries was at risk during the pandemic, placing women in a particularly vulnerable situation. Moreover, pre-existing differences in sexual pleasure (Andrejek and Fetner, 2019; Mahar et al., 2020), a higher susceptibility to affective disorders (Jalnapurkar et al., 2018) and to sexual dysfunction (Peixoto and Nobre, 2015), might have interacted with social isolation to decrease sexual health among women during this period. Most of all, changes in daily life, limitations on one’s own independence, and feelings of worthlessness, may have caused in women a sense of helplessness and loss (de Oliveira and Carvalho, 2021). The downturn in sexual satisfaction could not be avoided because of these COVID-19 prevention measures, which could lead to unrequited sexual needs, whilst also having a negative impact on one’s life satisfaction, especially for those who are sexually active, but not in a domestic partnership or marital relationship.

It is clear that many individuals’ sex lives have undergone a change, in which many have expanded their sexual repertoires to safeguard the own sexual satisfaction. Erwinda et al. (2020), found that changes with regard to sexual activity have prompted some people to compensate the non-fulfilment of their sexual needs, by, for example, accessing pornographic sites, by using dating apps and by entering into online sexual communication in order to express and satisfy their sexual desires.

Various studies observed that women reported a greater frequency of use of novel sexual activities for increasing sexual desire, sexual satisfaction and an increasing number of sexual outlets to cope with emotionally negative events (Cyranowski et al., 2004; de Oliveira and Carvalho, 2020; Lehmiller et al., 2020; Yuksel and Ozgor, 2020). These sexual outlets included activities such as trying out new sexual positions and sharing sexual fantasies with a partner, masturbation, reacting to erotic cues, cybersex, sexting (Lehmiller et al., 2020).

Sexting can be described as the creation and sharing of images or text messages in the personal sexual sphere, through the use of mobile phones or apps (Hasinoff, 2015), including Whatsapp, Facebook, Tinder, Snapchat, and other virtual applications. This has now become a basic element in most relationships, being helpful in beginning and maintaining a romantic connection (Albury et al., 2013; Cooper et al., 2016).

A growing number of studies have shown how sexting increased during the pandemic (Cocci et al., 2020; Lehmiller et al., 2020; Yuksel and Ozgor, 2020). About 15% of emerging adults started to engage in sexting during quarantine, as a result reporting greater satisfaction from their sex life (Lehmiller et al., 2020). The main benefit of sexting was to mitigate physical distancing (Fox and Potocki, 2014), because it allowed people to temporarily replace face-to-face interaction and satisfy their own needs in the sphere of sex. In fact, sexting represented an opportunity for maintaining intimacy and improving sexual satisfaction in a relationship (McDaniel and Drouin, 2015; Drouin et al., 2017).

The most widely reported reasons for sexting are connected with sexual and social aims (Bianchi et al., 2016, 2017). Within a relationship, people could “sext” with their own partners, by flirting, starting sexual activity or preserving a state of intimacy and passion (Temple and Choi, 2014; Van Ouytsel et al., 2017). Differently, for single people sexting could also be used as an excuse for attracting the interest of potential partners (Albury and Crawford, 2012). However, some studies found that sexting is a risk behavior. It can affect the physical and psychological health of people involved as well as trigger symptoms of psychological distress (Medrano et al., 2018). It could be associated to some risk behaviors, such as cyberpornography (Morelli et al., 2017), and online victimization which are associated to negative emotional impact (Slonje et al., 2017).

Garrido-Macías et al. (2021) examined some predictors that could be associated with sexting and they found that loneliness could lead to engagement in sexting by emerging adults. Other research demonstrates that high-stress conditions could negatively influence the functioning of a relationship (Burleson et al., 2007; Hamilton and Meston, 2013; De Witte et al., 2015; Lehmiller et al., 2020). Frequent engagement in various sexual activities has been associated with greater couple satisfaction among those in relationships; this effect may change in accordance with varying type and frequency of sexual activities, also influencing the intimacy and commitment of the couple (McNulty et al., 2016; Muise et al., 2016; Kort, 2020; Rosenberg et al., 2021). Furthermore, diverse studies indicate that couple intimacy, passion, and commitment with one’s own partner could reduce psychological distress and, thus, provide some relief in particular conditions, such as the current pandemic and its associated restrictions (De Witte et al., 2015; McNulty et al., 2016; Meston et al., 2020).

### The current research

As we have observed, sexual health is essential for one’s wellbeing. Some researchers observed how during the pandemic, activity linked to sexting provided psychological and emotional benefits (Erwinda et al., 2020; Lehmiller et al., 2020; Pennanen-Iire et al., 2021). Considering the difficulties women experienced in the sexual sphere during the pandemic, such as feelings of worthlessness and helplessness due social isolation (de Oliveira and Carvalho, 2021) emphasized by their higher susceptibility to affective disorders (Jalnapurkar et al., 2018) and to sexual dysfunctions (Peixoto and Nobre, 2015), it is very important to understand whether sexting could be a protective factor in women’s sexual and couple satisfaction. Obviously, these considerations are also important above and beyond the pandemic period.

The general aims of the present studies are: (1) to investigate the use of sexting among adult women during the isolation period of the second wave of COVID-19 pandemic, and exploring its coping function in the relationship between loneliness and sexual satisfaction, and (2) to analyze aspects of couple wellbeing of women who had engaged in sexting compared to those who had not.

## Study 1

**FIGURE 1:**
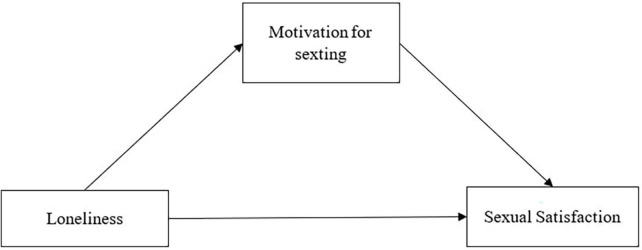
The hypothesized research model.

To date, the importance of sexting in sexual satisfaction in women during the COVID-19 crisis, but not only, has received little research attention, whereas it should be a research priority (de Oliveira and Carvalho, 2021). In the current investigation, we tested our hypothesized research model (Figure 1), in order to examine the relationship between loneliness, motivation for sexting and sexual satisfaction in women.

### The relationship between loneliness and sexual satisfaction

Loneliness and isolation might potentially prompt more sexual-negative-mood states, affecting sexual satisfaction and increasing relationship conflict (Lehmiller et al., 2020). Accordingly, quarantine situations may have exacerbated the worsening of sexual issues (Pennanen-Iire et al., 2021). Findings suggested that many women experienced a decrease in sexual satisfaction during the pandemic, due to the vulnerability factors that characterize the female population, including the consequences of forced isolation: cohabitation, a greater commitment to bringing up children, lower access to sexual health services (de Oliveira and Carvalho, 2021). In accordance with these studies, we hypothesize that:

H1: Loneliness was negatively related to sexual satisfaction for women during the second wave of the COVID-19 pandemic.

### The relationship between sexting and sexual satisfaction

Sexual satisfaction is considered the affective response resulting from an individual’s subjective estimation of the positive and negative dimensions in his/her own sexual sphere (Lawrance and Byers, 1995). Past research has shown that there is a relationship between sexting and sexual satisfaction, considering sexting as a way of sexual communication and also as sexual behavior (Stasko and Geller, 2015; Galovan et al., 2018). Specifically, it has been seen that sexual satisfaction increases as the frequency of sexting with one’s sexual partner increases. Thus, it has been shown how sexual satisfaction can be enhanced through sexting as it can function both as a strategy of communication and activity linked to the sexual sphere (Oriza and Hanipraja, 2020). Starting from this literature, we hypothesize that:

H2: Motivation for sexting was positively related to sexual satisfaction for women during the second wave of the COVID-19 pandemic.

### The mediating role of motivation for sexting

Previous studies noted a decrease in actual live communication with other people; an increase in isolation and loneliness have been linked with greater use of the Internet for sexual purposes (Kraut et al., 1998; Barak and Fisher, 2002). Specifically, prior research showed that loneliness could lead to engagement in sexting by emerging adults (Garrido-Macías et al., 2021). During confinement due to the pandemic, it was shown how loneliness and isolation could potentially prompt increased sexual adaptation in order to fulfill sexual needs or relieve negative mood states, affecting sexual satisfaction (Lehmiller et al., 2020). Engaging in sexting may therefore reflect a coping strategy to combat a sense of isolation or an intentional strategy for preventing further sexual restrictions, so we hypothesize that:

H3: Motivation for sexting mediated the negative effects of loneliness and sexual satisfaction for women during the second wave of the COVID-19 pandemic.

### Method

#### Participants and procedure

A convenient sample of university students took part in the study for a credit course. The only condition was: (a) that they were women; (b) that they had been involved in a romantic relationship (but not cohabiting) for at least a year; (c) that they declare that they had been sexting during the pandemic. In the end, three hundred and twelve women (mean age = 26.98, SD = 6.98; age range = 20–51) participated in the study. The administration of the questionnaire took place online between 15 November 2020 and 15 March 2021 (during the second wave of the COVID-19 pandemic in Italy). The questionnaire was anonymous and took approximately 10–15 min to be completed. The research was conducted in accordance with the ethical standards of the Italian Psychological Association (AIP), as well as the Declaration of Helsinki. All participants completed statements of informed consent to participate in the study.

#### Measures

The following measures were used to achieve the objective of the study.

#### Information about relationship status and sexting

Participants were asked to answer three questions regarding relationship status and sexting. Particularly, the questions explored the following: (1) being in a couple relationship for at least 1 year; (2) the type of relationship (homosexual or heterosexual); (3) after a brief introduction on sexting behavior, if she had practiced sexting with her partner at least once during the last 6 months of restrictions caused by the COVID-19 pandemic.

##### Loneliness

Measured using the UCLA Loneliness Scale–Version 3 (Russell, 1996), a global loneliness self-report measure composed of 20 items (e.g., “How often do you feel alone?”, “How often do you feel that people are around you but not with you?”). The scale is evaluated on a 4-point Likert-type scale, from 1 = Never to 4 = Often with higher scores indicating greater tendencies to feel lonely (α = 0.90).

##### Motivation for sexting

Assessed with the 13-item self-report Sexting Motivations Questionnaire (SMQ; Bianchi et al., 2016), which measures the frequency of three motivations for sexting: body image reinforcement (3 items; e.g., “Sometimes I send sexts to test whether I am attractive enough”), sexual purposes (5 items; e.g., “Sometimes I send sexts to increase passion in my dating relationship”), and instrumental/aggravated reasons (5 items; e.g., “Sometimes I send sexts in exchange for money or gifts”). Answers included a 5-point scale (from 1 = Never to 5 = Always) (α = 0.91). In the present study, we used an indicator of *motivation for sexting* that included only the dimensions of body image reinforcement and sexual purposes, excluding instrumental/aggravated for theoretical reasons connected with the objectives of the study that considers sexting as a coping strategy.

##### Sexual satisfaction

Measured using the New Sexual Satisfaction Scale short form (NSSS-S; Brouillard et al., 2019), 12 items were rated on a 5-point Likert scale, from 1 = Not at all Satisfied to 4 = Extremely Satisfied with higher scores indicating higher levels of sexual satisfaction. As an example of items there was “The way I sexually react to my partner” and “My partner’s emotional opening up during sex” (α = 0.94).

### Results

Preliminary analyses revealed no substantial violation of normality regarding data distribution. Means and standard deviations are listed in Table 1.

**TABLE 1:** Means, standard deviation and correlations between loneliness, motivation for sexting and sexual satisfaction.

	M	SD	Skewness	Kurtosis	1	2	3	4
1. Age	26.98	6.98	1.51	1.84	−			
2. Loneliness	2.26	0.52	0.154	-0.377	-0.107	−		
3. Motivation for sexting	2.52	0.90	0.075	-0.338	-0.105	-0.262**	−	
4. Sexual satisfaction	3.74	0.92	-0.930	0.646	0.015	-0.314**	0.384**	−

The data collection of this study was conducted between 15 December 2020 and 15 March 2021.

**p < 0.01.

Analyzing correlation patterns, we can see how loneliness and sexual satisfaction are negatively correlated as we hypnotized in H1. Motivation for sexting is also positively related with sexual satisfaction: those who scored higher in motivation for sexting were also more sexually satisfied, as previously assumed (H2) (Table 1).

In order to test the third hypothesis, we tested the role of the mediator of motivation for sexting in the relationship between loneliness and sexual satisfaction. To test our hypothesis we used PROCESS model 4 (5,000 resampling; see Figure 1).

**FIGURE 2:**
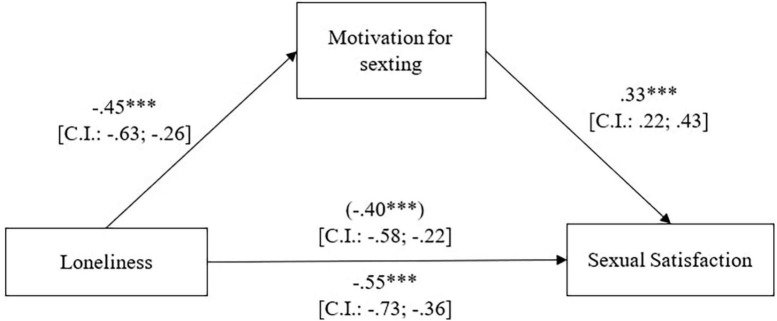
The effects of loneliness on sexual satisfaction *via* motivation for sexting.

The overall equation was significant, *R*^2^ = 0.10, [*F*(1,310) = 33.93, p < 0.001]. As we can see in Figure 2, loneliness significantly predicted both motivation for sexting and sexual satisfaction in a negative way. Sexual satisfaction is also predicted by motivation for sexting. It is also possible to observe how the indirect effect of the outcome of loneliness on sexual satisfaction through motivation for sexting was significant.

### Discussion

Study 1 shed light on the use of sexting among adult women during the second wave of the COVID-19 pandemic and explored its coping function in the relationships between loneliness and sexual satisfaction.

As regards gender, past literature suggests that women would be less prepared to engage in sexting behavior, due to prevailing sexual mores, according to which women feared much heavier social consequences than men (Ringrose et al., 2013; Salter, 2016; Setty, 2019), but it was shown that, during the pandemic, this bias was not found in the female sample. For example, Thomas et al. (2022) found no effect of gender on willingness to engage in sexting, probably due to sexual needs that had arisen in the wake of COVID-19 restrictions. Recent studies indicated an increase in sexting activities during the first phase of the pandemic (Gabster et al., 2022), suggesting that it could have had a role in coping with pandemic difficulties, such as sexual and couple satisfaction. However, to our knowledge, this research provides the first evidence about the role of sexting in tackling pandemic-related loneliness and its influence on sexual satisfaction.

The results of this study showed that the model of the links between loneliness and sexual satisfaction (through the mediating role of motivation for sexting) has a good fit, underlining the predicting role of sexting and loneliness in sexual satisfaction. The study also emphasized the role of motivation for sexting as a mediator in the relationship between loneliness and sexual satisfaction. In detail, we observed a negative relationship between loneliness and sexual satisfaction, which is consistent with the results of research by Van Ouytsel et al. (2017) and Abedi et al. (2020). These previous results emphasize that higher levels of loneliness are predictive of lower sexual satisfaction. When we introduced motivation for sexting as a mediator we found an indirect relationship between loneliness and sexual satisfaction; specifically, loneliness negatively predicted motivation for sexting, which in turn positively predicted sexual satisfaction. Indeed, during the pandemic, it was seen how having a steady partner and, therefore, not feeling lonely was a good predictor of engagement in sexting, used as a coping strategy aimed to reduce perceived distance and increase sexual satisfaction (Caponnetto et al., 2022). It is plausible that the increase in the use of technology-based sexual activity such as sexting, during the pandemic, was a temporary coping strategy that would subsequently be replaced in favor of future in-person interaction with one’s own partner at the end of the pandemic (Lehmiller et al., 2020).

Sexting has been shown to be an adaptive coping response to pandemic sexual difficulties in other recent research (Bianchi et al., 2021a), suggesting that sexual satisfaction was the result of sexting applied as an antidote to pandemic worries. Caponnetto et al. (2022) also found that during the pandemic the perceptions of sexting changed, and this modification seems to be linked to the pandemic socio-sanitary situation and related restrictions. These results are in disagreement with some previous findings, which indicated that cybersex activities like sexting, were significantly connected with a decrease in marital sexual satisfaction and intimacy (Whitty, 2003), probably because under normal conditions people perceived online sexual activity as menacing couple fidelity (Cooper et al., 2006).

In fact, a pandemic study on adults by Lehmiller et al. (2020) showed that 20.3% of the sample reported new sexual activities in their sexual life during the pandemic, e.g., sharing nude photos, sexting, having cybersex, filming oneself masturbating, and using advanced sexual technology such as virtual reality porn. In addition, using sexting behavior as a coping strategy during COVID-19 affected the sexuality of people in different ways (Bianchi et al., 2021a), because some people found themselves reinventing their ways of experiencing their own sexuality (Eleuteri et al., 2022). For these reasons, it becomes critical to explore people’s sexual lives at the time of COVID-19 by investigating changes in sexual behavior patterns since the pandemic began until its current evolution.

## Study 2

Results from Study 1 showed how sexting played an important role in the mediation of the relationship between loneliness and sexual satisfaction in conditions of social isolation due to the COVID-19 pandemic. The women who engaged in sexting in conditions of isolation from their partner also showed higher levels of sexual satisfaction, confirming other research that found that sexual interest was enhanced by trying out new fantasies, modes of foreplay, sexual positions, and digital sex, leading to probable improvements in their sex lives (Lehmiller et al., 2020).

Other research showed how changes due to social isolation during COVID-19 generated many changes, all over the world, in the couple relationship, especially in terms of couple stability (Schmid et al., 2021), well-functioning intimate relationships (Luetke et al., 2020), couple wellbeing (Oriza and Hanipraja, 2020), and general satisfaction (Bolesławska et al., 2021).

Considering that sexual satisfaction is an extremely important component of a couple’s life (Sánchez-Fuentes et al., 2014; Flynn et al., 2016), it is possible then that sexting might also influence other aspects of the relationship, generating levels of general satisfaction. Study 2 sought to investigate these aspects in more detail.

Following these considerations, we looked more deeply at the relationship analyzed in Study 1 and observed other aspects of couple wellbeing (intimacy, passion, commitment, and general couple satisfaction) in women who had been sexting during the pandemic compared to those who had not. Therefore, we hypothesize that:

H1: Couple wellbeing (intimacy, passion, commitment, and couple satisfaction) is greater among women who had been sexting during the pandemic compared to those who had not.

Also, previous research observed how women are more preoccupied than men about chatting freely with another person, and as a result they feel more jealous of their own partner (Weinstein et al., 2015). These results were also confirmed during the pandemic period, in which Shafer et al. (2022) found that COVID-related health concerns predicted greater social media surveillance of a romantic partner during the COVID quarantine. Surveillance is characterized of jealousy and it is typical of people who try to safeguard themselves from potential threatening circumstances (Tokunaga, 2016). In the Internet context, surveillance is easier and it is related to supervise romantic partners’ wall updates, postings, friends and followers lists, videos, photos, and even invitations, because the supervising behavior could help oneself to diminish relational uncertainty (Marshall et al., 2013). Starting from this literature, we also hypothesize that:

H2: Electronic surveillance is greater among women who had been sexting during the pandemic compared to those who had not.

### Method

#### Participants and procedure

Three hundred and forty-two women (*M* = 23.05; SD = 3.20; age range = 19–34) who had been involved in a romantic relationship for at least 1 year were recruited in order to complete an online survey. Participants were university students and did not receive any compensation for their participation. The administration took place between 15 February 2021 and 15 March 2021 (during the final phase of the second wave of the pandemic in Italy). The data collected were anonymous and all participants provided written informed consent. Participants were informed about the aim of the research during the debriefing. All procedures performed in this study were in accordance with the ethical standards of the Italian Psychological Association (AIP) and with the Helsinki Declaration.

Participants had to answer the first item of the questionnaire by declaring whether they had ben sexing during the pandemic (“Did you practice sexting at least once during the last 4 months of restrictions caused by COVID-19 pandemic?”). Two hundred and three women declared that they had not been sexting during the pandemic (WHNS), one hundred and thirty-nine women declared that they had been sexting at least once (WHS). Eleven participants declared they were in homosexual relationships.

#### Measures

##### Information about relationship status and sexting

Participants were asked to answer three questions regarding relationship status and sexting. Particularly, the questions explored the following: (1) being in a couple relationship for at least 1 year, because when the research was conducted only the last year corresponded to the pandemic period; (2) the type of relationship (homosexual or heterosexual); (3) if she had practiced sexting with her partner at least once during the last 4 months of restrictions caused by the COVID-19 pandemic.

##### Intimacy, passion, and commitment

The Triangular Love Scale (Sternberg, 1988, 1997) was used to assess three components of love toward the romantic partner: intimacy (15 items; α = 0.95; e.g., “I am willing to share myself and my possessions with…”), passion (15 items; α = 0.94; e.g., “I especially like physical contact with …”), and commitment (15 items; α = 0.93; e.g., “I cannot imagine ending my relationship with…”). Respondents were asked to think about the person they love and rate the agreement on a 9-point Likert scale, from 1 (Not at all) to 9 (Extremely).

##### Couple satisfaction

Measured using the ENRICH (Evaluation and Nurturing Relationship Issues, Communication and Happiness) Marital Satisfaction Scale (EMS; Fowers and Olson, 1993). For the purpose of this study 6 items have been used (e.g., “My partner and I understand each other perfectly”). The scale is evaluated on a 5-point Likert-type scale (α = 0.75), from 1 (strongly disagree) to 5 (strongly agree) with higher scores indicating a greater positive couple agreement.

##### Electronic surveillance

Assessed using the Interpersonal Electronic Surveillance Scale for Social Network Sites (ISS; Tokunaga, 2011), a 12 item self-report scale which measures the interpersonal electronic surveillance over SNSs (e.g., “I explore my partner’s social networking page to see if there is anything new or exciting”). The scale is evaluated on a 7-point Likert-type scale, from 1 (strongly disagree) to 7 (strongly agree) (α = 0.94).

### Results

Preliminary analyses revealed no substantial violation of normality regarding data distribution. Means and standard deviations for both groups are listed in Table 2.

**TABLE 2:** Mean and standard deviation of the dependent variables on women who declare that they had not sexting during pandemic (WHNS), and women declared that they had sexting at least once (WHS).

	WHS				WHNS			
	**M**	**SD**	**Skewness**	**Kurtosis**	**M**	**SD**	**Skewness**	**Kurtosis**
Intimacy	7.53	0.79	-0.574	0.394	7.03	0.96	-0.548	0.143
Passion	7.62	0.81	-0.405	-0.646	7.00	0.97	-0.219	-0.384
Commitment	7.15	0.86	0.163	-0.592	7.17	0.85	-0.272	-0.398
Satisfaction	4.29	0.45	-0.458	-0.416	4.02	0.49	-0.132	-0.875
Electronic surveillance	4.75	1.22	-0.226	-0.385	4.07	1.28	0.462	-0.237

We initially conducted a MANOVA to detect the presence of multivariate effects associated with engagement (or not) in sexting with her partner during the lockdown period. The results showed the presence of a significant effect [Wilks’ λ(5,336) = 20.51, *p* < 0.000, partial η^2^ = 0.23] (Table 2).

At the univariate level, to test our hypotheses, we ran five ANOVAs comparisons for each of the dependent variables (intimacy, passion, commitment, couple satisfaction, electronic surveillance) between women who declared that they had not been sexting during the pandemic (WHNS), and women who declared that they had been sexting at least once (WHS).

The results show that there are differences between the two groups with regard to the Triangular Love Scale (Sternberg, 1988, 1997). In particular, differences emerge regarding the levels of intimacy [*F*(1,340) = 25.51; *p* < 0.001, partial η^2^ = 0.07] and passion [*F*(1,340) = 37.75; *p* < 0.001, partial η^2^ = 0.10] (greater levels of intimacy and passion for WHS), but no difference emerges regarding the levels of commitment [*F*(1,340) = 0.89; *p* = n.s.] (Table 2). Also, with regard to the levels of couple satisfaction we observed a difference between WHS and WHNS [*F*(1,340) = 25.51; *p* < 0.001, partial η^2^ = 0.70]. In particular, women who engaged in sexting show higher levels of couple satisfaction.

Finally, we found that WHS also activate higher levels of partner surveillance than WNHS [*F*(1,340) = 24.15; *p* < 0.001, η^2^ = 0.07].

### Discussion

The results of Study 2 confirmed the important role of sexting as a resource for combatting social isolation in women due to the pandemic condition. The results highlight that women WHS during the second wave of the pandemic, compared to those WHNS, had higher scores on intimacy, passion, couple satisfaction and electronic surveillance. These findings support previous evidence for sexting activities affecting couple wellbeing (McDaniel and Drouin, 2015; Oriza and Hanipraja, 2020).

More in detail, previous research has observed that intimacy is an important dimension of cybersex. Weinstein et al. (2015) found that women prefer to engage in online sexual activity to increase the couple’s intimacy. The primary reason for using online sexual activities was as a part of lovemaking with one’s own partner or in response to requests by their partner, since most women interpret online sexual activities as acceptable or positive when associated with a shared activity with a partner. These results are also confirmed by Caponnetto et al. (2022), who observed that, during the pandemic, the perception of sexting improved, and was used as a coping strategy and a way of reducing the perceived distance with one’s partner. Other studies confirmed an increase in sexting during the pandemic (Bianchi et al., 2021a; Eleuteri et al., 2022) and investigated the relationship between COVID-related stress, coping strategies, and experimental, risky, and emotional sexting. They found that, during quarantine, for those who were in a long-distance relationship as opposed to those who were in a non-distance relationship or single, as an attempt to maintain intimacy with partners, there was a high frequency of experimental sexting–a type of sexting used when sexual contents are shared with a trusted partner (Wolak et al., 2012; Eleuteri et al., 2017).

In Study 2, we also observed that women who engaged in sexting during the isolation period reported higher levels of passion and couple satisfaction. Previous research (Mollaioli et al., 2021) found that those who had had more sexual activity during the pandemic showed a decrease in psychological distress and a higher level of dyadic commitment and couple satisfaction, compared with those who did not. In this vein, sexual activity seems to perform a protective role on one’s personal psychological health. In general, improvements in sexuality were associated with an improvement in the relationship with the partner, being happy and satisfied together, feeling less stressed (Arafat et al., 2020; Panzeri et al., 2021; Costantini et al., 2021). Furthermore, in a large study conducted by Balzarini et al. (2023), perceived virtual partner responsiveness helped to improve poor relationship quality associated with pandemic-related stressors. Ouytsel et al. (2019) found that engagement in sexting with one’s own partner was significantly linked to a higher perception of passion within the relationship.

In our results, no difference emerges regarding the levels of commitment between women WHS during the second wave of the pandemic compared to the WHNS. Contrary to our expectations, but in line with Matotek et al. (2021), this result is probably produced by basal high rates of couples’ commitment in our sample, for which sexting could simply constitute technology-mediated communication, useful for the couple’s more passionate and sexual aspects.

Previous research has shown how women often report that cybersex in general is characterized by chatting freely with another person, and as a result women feel more jealous than men about this activity on the part of their partner (Weinstein et al., 2015; Ruggieri et al., 2021b). This is confirmed by the results of this study, which suggest that WHS during the second wave of the pandemic, compared to WHNS, had higher scores on electronic surveillance. A reason for greater surveillance could be interpreted as the belief that one’s partner, during the period of isolation, is more inclined to also sexting with other women, being less likely to be discovered due to the forced separation caused by the pandemic. The same results were found by Shafer et al. (2022) in a cross-lagged study on adults, finding that COVID-related health concerns predicted social media surveillance of a romantic partner during COVID quarantine. Although romantic partners report developing a sense of closeness and intimacy when sexting, it is important to bear in mind how feelings of distress and uncertainty by adult couples have been shown to lead to social media surveillance of current partners (Fox and Warber, 2014) and this may have been intensified during the COVID-19 pandemic (Goodboy et al., 2021).

## Conclusion

During the pandemic, women underwent a deterioration in their sexual functioning, with a decline in sexual satisfaction (de Oliveira and Carvalho, 2021). Eleuteri et al. (2022) found that anxiety, stress, and depressive symptoms due to the pandemic influenced this decrease in sexual desire for women. As a result, the frequency of sexting also increased during the pandemic (Lehmiller et al., 2020). Although previous research has shown the role of sexual activities based on technology usage and how these allowed one to remain connected during social distancing, to our knowledge there is no research on women’s perceived couple wellbeing associated with sexting during the pandemic. Compared with previous studies conducted during the COVID-19 pandemic period (Bianchi et al., 2021b; Caponnetto et al., 2022; Eleuteri et al., 2022), our study focuses on the role of sexting as an adaptive coping response combatting loneliness and social isolation and promoting sexual satisfaction and couple wellbeing in women in response to pandemic-based sexual difficulties.

Our results demonstrate the role of the online dimension to surmount physical distancing and isolation under the particular restrictions, despite the negative consequences that sexting could generate in some cases that should not be underestimated. Instead, our findings contribute to better understand positive aspects of sexting; the fact that people enjoy improved sexual satisfaction through sexting, and that sexting is linked to increased couple wellbeing, suggests that this behavior could carry out a psychological function. Specifically, these results may indicate that engagement in sexting may serve as an adaptive coping mechanism during particularly difficult circumstances.

Taken together, these results suggest that during the epidemic, engagement in sexting for women may have a protective role in safeguarding couple wellbeing.

### Limitations and future research

Study limitations when interpreting results should also be considered. The first limitation of this study is that our results are based on correlational data, and great caution should be made in interpreting them causally. Also, the literature suggests that other variables belonging to the individual’s personality could be implicated in sexting behavior, such as openness to new experiences, machiavellianism or narcissism (Crimmins and Seigfried-Spellar, 2017; Morelli et al., 2021). Further research should investigate the relationship between sexting and other variables and should be conducted mainly with experimental and longitudinal approaches, to clarify the direction of these relationships and understanding potential reciprocal relationships between the variables. The second limitation of the research is that it was conducted in Italy during the second wave of the COVID-19 pandemic, and the results cannot be generalized to different countries, where the experience of the second wave of the pandemic may have been very different. Further research conducted in other cultural contexts (also in the absence of the restrictions produced by the COVID-19 pandemic) should investigate the relationship between sexting and individual wellbeing in women. The third limitation is linked to the sample, because the participants are only women university students in a specific type of relationship, over 1 year and not cohabiting. Further research should investigate the relationship between sexting and individual wellbeing in different types of samples such as men and general population (instead of young students only). Another limitation is the adoption of self-report instruments, which can be influenced by the social desirability bias; they may also limit conclusions from these results. Finally, we only assessed women, and it might be interesting to understand how the relationship between these variables would work in a sample of men as well. Future research needs to focus on a longitudinal design and add objective assessments of variables.

Despite these limitations, our study may bring something fresh to research, providing the first evidence for the coping role of sexting behaviors in sexual satisfaction and couple wellbeing. Our findings should be used in post-pandemic studies to better understand the coping function of sexting behavior in other environmental conditions, beyond the pandemic. Since the view of technology-mediated sexuality is at a developmental stage, future work should investigate the sexting role in sexual satisfaction with a longitudinal design, to understand how it could increase the level of intimacy within a relational relationship. From a clinical point of view, we believe that the results of our work may be useful in better understanding the phenomenon of sexting during emergencies, of sexual interactions mediated by technology, and the impact on sexual satisfaction for women.

## Data availability statement

The raw data supporting the conclusions of this article will be made available by the authors, without undue reservation.

## Ethics statement

The studies involving human participants were reviewed and approved by the University of Palermo. The patients/participants provided their written informed consent to participate in this study.

## Author contributions

RB, MG, and GL performed the material preparation and data collection. SR performed the analyses. RB and SR wrote the first draft of the manuscript. All authors contributed to the study conception and design and commented on previous versions of the manuscript, read, and approved the final manuscript.
